# Specimen Collection and Confirmation of Norovirus Outbreaks^1^

**DOI:** 10.3201/eid1708.101815

**Published:** 2011-08

**Authors:** Melissa S. Plantenga, Beletshachew Shiferaw, William E. Keene, Christianne Biggs, James M. Terry, LaDonna Grenz, Paul R. Cieslak

**Affiliations:** Author affiliation: Oregon Department of Human Services, Portland, Oregon, USA

**Keywords:** Norovirus, outbreaks, stool specimens, diarrhea, viruses, dispatch

## Abstract

We evaluated data from gastroenteritis outbreaks in Oregon to assess sensitivity of stool testing for norovirus and determine number of specimens needed to confirm norovirus as the cause. Norovirus can be readily confirmed if 3–6 specimens are collected any time <7 days after onset of diarrhea and for almost that long after symptoms resolve.

One goal of any outbreak investigation is to identify the causative pathogen (*1*). In a recent analysis of foodborne disease outbreaks in the United States during 2006, only 49% had a confirmed causative pathogen (*2*). A review of foodborne disease outbreaks investigated in the 10-site Foodborne Disease Active Surveillance Network during 1998–1999 found that an etiologic agent was identified for only 29% (*3*). The major limitation in identifying etiologic agents was a lack of specimens; no stool specimens were collected in two thirds of the unconfirmed outbreaks.

In Oregon, outbreaks of illness are reportable to public health authorities, who investigate to determine the causative pathogen and means of transmission and to implement control measures accordingly. Obtaining stool specimens within 3 days of onset has been recommended (*4*), but carrying out this recommendation is frequently not feasible.

Since 1999, specimens from case-patients in outbreaks of acute gastroenteritis have been tested for norovirus at the Oregon State Public Health Laboratory. Noroviruses are a group of related, nonenveloped, single-stranded RNA viruses that cause acute gastroenteritis in humans. We reviewed Oregon data to assess the sensitivity of stool testing for norovirus at different times after illness onset and to determine the number of specimens needed to ensure a high probability of confirming a norovirus outbreak.

## The Study

We reviewed all outbreaks of acute gastroenteritis reported in Oregon from August 23, 1999, through January 31, 2007, for which norovirus was the suspected cause on the basis of incubation period, symptoms, and duration of illness. As part of the outbreak investigations, demographic, clinical and exposure information was gathered, and specimens were solicited with the goal (often unmet) of obtaining at least 3 specimens per cluster. Public health investigators were exhorted to collect specimens “as soon as possible” after a cluster was identified. From 1999 until November 2005, specimens were tested by conventional reverse transcription PCR (RT-PCR) by using consensus primers for norovirus (*5*). From November 2005 onward, specimens were tested by real-time RT-PCR by using primers to a highly conserved region in the open reading frame 1–2 junction (*6**,**7*). Laboratory results were linked to epidemiologic and clinical information ascertained during the outbreak investigation. We defined a confirmed norovirus outbreak as a cluster of compatible illnesses for which norovirus sequences were identified from specimens of at least 2 case-patients (*8*).

**Figure 1:**
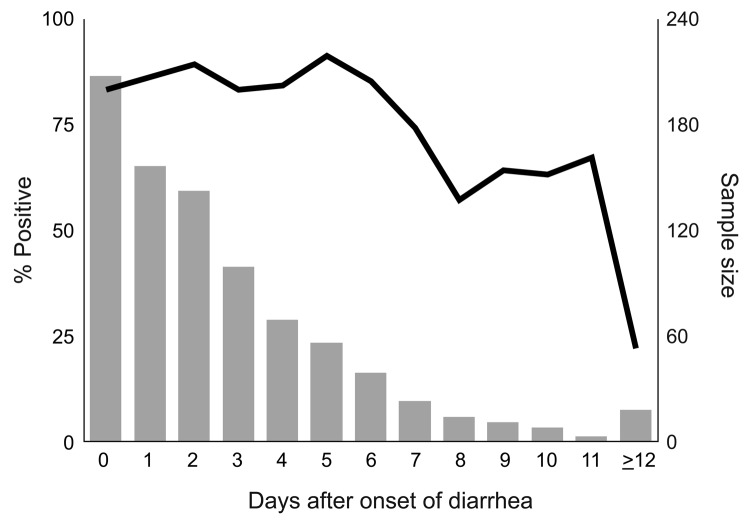
Percentage of specimens positive for norovirus (line), by days after onset of diarrhea, Oregon, USA, August 1999–January 2007.

From August 23, 1999, through January 31, 2007, a total of 486 (59%) of 824 reported outbreaks of acute gastroenteritis in Oregon were suspected to be caused by norovirus. Norovirus was confirmed as the cause of 355 outbreaks; of these reports, 275 (77%) provided analyzable data. The outbreaks were associated with the following settings: 151 (55%) with nursing homes, long-term-care facilities, or assisted-living centers; 41 (15%) with restaurants or delicatessens; 10 (4%) with schools; 11 (4%) with hospitals; 8 (3%) with private homes; 6 (2%) with camps; 4 (1%) with correction facilities; and 44 (16%) with other settings. From these 275 outbreaks, 1,117 specimens were submitted to the laboratory with a median of 4 (range 2–13) per outbreak; 888 (79%) tested positive for norovirus. Of the 1,117 specimens, dates of diarrhea onset and stool collection were available for 845 (76%). Of those, 698 (83%) tested positive for norovirus. Figure 1 depicts the percentage of specimens that tested positive, by time since diarrhea onset.

Of the 1,117 specimens sent to the laboratory, dates on diarrhea resolution and stool collection were available for 360 (32%) in 153 outbreaks. Of those, 302 (84%) tested positive for norovirus. No association was found between PCR positivity and patient sex (relative risk for male patients 1.06; p = 0.07) or age.

**Figure 2:**
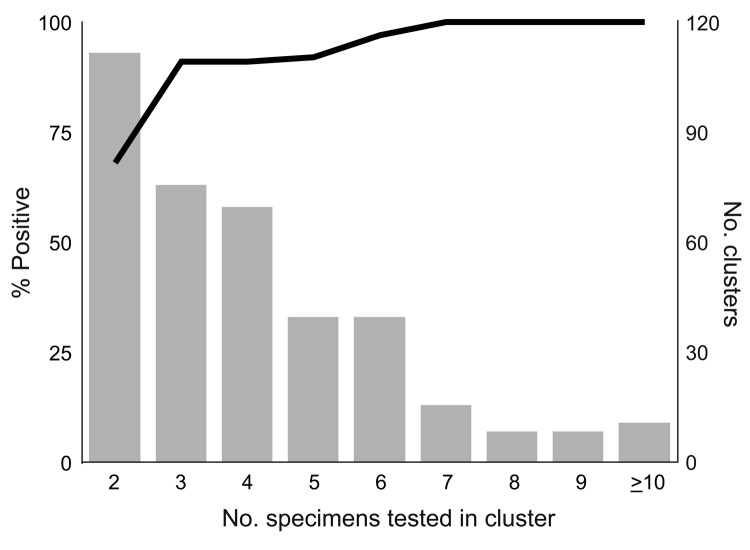
Percentage of outbreaks confirmed as norovirus (line), by number of specimens tested, Oregon, USA, August 1999–January 2007.

To evaluate the number of stool specimens needed to confirm norovirus as the etiologic agent of an outbreak (suspected to have been caused by norovirus), we analyzed 377 such outbreaks from the same period. A total of 1,532 specimens from these outbreaks were tested, and norovirus was identified in 1,134 (74%). Figure 2 shows likelihood of an outbreak being confirmed as caused by norovirus as a function of the number of specimens tested.

## Conclusions

Our data indicate that 3 specimens were sufficient to confirm 91% of norovirus outbreaks, and when 6 specimens were submitted, 97% were confirmed. Testing >7 specimens added nothing to the sensitivity. Therefore, we recommend that to confirm that norovirus is the etiologic agent of an outbreak, at least 3, but not more than 6, specimens should be collected. Norovirus can be readily confirmed as the cause of an outbreak of acute gastroenteritis if specimens are collected at any time during the first 7 days after onset of diarrhea and (though period is somewhat more variable) for almost that long after symptoms resolve. No apparent decline in sensitivity occurs for specimens collected up to 6 days after onset of diarrhea. Sensitivity drops during days 7–14, but remains substantial for up to 2 weeks. The common excuse that “I’ve already gotten better” can be safely ignored. Several studies have examined the duration of norovirus excretion and found that the average period of shedding is ≈28 days (range 13–56 days), well past the resolution of symptoms (*9**,**10*). Infectivity cannot be inferred from these findings, however.

These data have several limitations. First, at nursing homes, the responsibility for data collection was often delegated to staff, and no attempt was made to validate the data they submitted. Second, data were incomplete for many cases. Third, few specimens were collected >14 days after diarrhea onset or >7 days after diarrhea cessation, yielding wider confidence intervals around late-sample point estimates. Lastly, a real-time RT-PCR is now being used by the Oregon State Public Health Laboratory (and many others); this assay is up to 10,000× more sensitive than the conventional RT-PCR (*11*), and the number of specimens needed to confirm norovirus outbreaks may decrease as the acceptable time range for collection increases. We noted a trend toward higher positivity rates with the real-time RT-PCR, so fewer specimens might be required; norovirus was confirmed in 100% of outbreaks with >5 specimens tested by this method. Despite these limitations, we believe that this information will be helpful to outbreak investigators and to researchers studying sporadic cases of acute gastroenteritis. Taking into account these findings, in turn, will lead to a more accurate picture of the incidence of norovirus infections.
